# Advances in the treatment of transthyretin amyloidosis

**DOI:** 10.1136/egastro-2025-100198

**Published:** 2025-07-18

**Authors:** Intissar Anan

**Affiliations:** 1 Department of Public Health and Clinical Medicine, Umeå University, Umeå, Sweden; 2 Department of Medicine, Umeå University Hospital, Umeå, Sweden

**Keywords:** Gastroenterology, Genes, Genetics, Liver Diseases, Liver Transplantation, Amyloidosis, Hereditary, Transthyretin-Related

## Abstract

This review aims to provide a comprehensive overview of the existing therapeutic options for managing neuropathic and/or cardiac manifestations associated with transthyretin amyloidosis (ATTR), along with investigational therapeutic candidates under evaluation in ongoing clinical trials. Additionally, emerging approaches for combating this life-threatening disease are discussed. Recent advancements in non-invasive diagnostic techniques for the detection of ATTR have facilitated improved diagnosis and identification at an earlier disease stage, thereby enhancing the potential efficacy of therapeutic interventions. Presently, there exists a range of clinically available treatments targeting ATTR, alongside investigational agents undergoing assessment in clinical trials. Therapeutic modalities encompass tetramer stabilisation, gene silencing, and ATTR fibril disruption and removal strategies. Historically, ATTR has been underdiagnosed. However, with the progression of diagnostic methodologies and the introduction of disease-modifying treatments, early diagnosis and initiation of treatment have significantly transformed the management of this condition, and effective treatment modalities have been introduced and are under development.

## Introduction

### Biology of transthyretin

 Transthyretin (TTR) is a tetrameric plasma protein primarily synthesised by hepatocytes in the liver.^1^ It plays a key role in the transport of thyroxine (T4) and retinol-binding protein bound to vitamin A.^1^ Smaller amounts of TTR are also produced in the choroid plexus of the brain and the retinal pigment epithelium in the eye.^2^ More than 220 mutations in the *TTR* gene have been identified, many of which are associated with hereditary forms of amyloidosis.^3 4^

### Pathogenesis of transthyretin amyloidosis

TTR amyloidosis occurs when the TTR tetramer dissociates into monomers, which then misfold and aggregate into insoluble amyloid fibrils.^5^ This dissociation is considered the rate-limiting step in the pathogenesis. The misfolded monomers undergo conformational changes, exposing hydrophobic regions that promote β-sheet formation and oligomerisation.^5^ These oligomers eventually aggregate into amyloid fibrils that deposit extracellularly in tissues such as the peripheral nerves, myocardium, gastrointestinal tract, kidneys and eyes.^6^ These deposits disrupt tissue architecture and function, leading to organ dysfunction and clinical symptoms.^6^

Mutations in the *TTR* gene destabilise the tetramer structure, accelerating the aggregation process. However, even wild type TTR can form amyloid fibrils with ageing, leading to the non-hereditary form of the disease.^7^

### Hereditary transthyretin amyloidosis

Hereditary transthyretin amyloidosis (ATTRv) is a rare autosomal dominant disorder characterised by systemic deposition of amyloid fibrils composed of mutant TTR.^8^ Clinical manifestations vary based on the mutation but commonly include peripheral neuropathy, autonomic dysfunction and cardiomyopathy (CM).^9^ The age of onset and disease progression are influenced by genetic and environmental factors.

The prevalence of ATTRv varies geographically and is influenced by the distribution of specific TTR mutations within different populations.^8 10^ Certain mutations are more prevalent in various regions, leading to regional clusters of the disease.^11^ The most common TTR mutation associated with ATTRv is the Val30Met (V30M) mutation, which has been identified worldwide but especially in the Portuguese, Japanese and Swedish populations.^12^,^14^

ATTRv typically manifests clinically in mid-adulthood, although the age of onset can vary widely depending on the specific TTR mutation and other genetic and environmental factors. Clinical manifestations of ATTRv are heterogeneous and can include neurological symptoms^15^ (such as peripheral neuropathy and autonomic dysfunction), cardiac involvement^15^ (including CM and arrhythmias), ocular manifestations^16^ (such as vitreous opacities and glaucoma), gastrointestinal symptoms^17^ and renal involvement.^18^ In a few rare mutations, central nervous system manifestations predominate.^19^

The prognosis of ATTRv is generally poor, with progressive deterioration in organ function leading to significant morbidity and mortality.^20^ The disease course can vary widely between individuals, with some patients experiencing rapid progression and others exhibiting a more indolent course. Early diagnosis and intervention are crucial for optimising patient outcomes and quality of life (QOL).^20^

### Wild type transthyretin amyloidosis

Wild type transthyretin amyloidosis (ATTRwt), formerly known as senile systemic amyloidosis, is a disease of the elderly population and predominantly affects men over the age of 65 years.^21^ The disease is characterised by progressive congestive heart failure that is caused by deposition of amyloid in the heart, leading to impaired myocyte function and predominantly diastolic heart failure.^22^

The prognosis of ATTRwt is poor when the patient has developed heart failure, with a suspected survival of 3–4 years.^23^ Until the advent of disease-modifying therapies, treatment options were limited to supportive care, with heart transplantation rarely feasible due to advanced age and comorbidities in most patients.

### Epidemiology and global disease burden

Once considered rare, transthyretin amyloidosis (ATTR) is now recognised as more prevalent, largely due to improved diagnostic methods. ATTRv is estimated to affect 5000–38 000 individuals globally, though actual prevalence is likely higher due to underdiagnosis.^24^ Endemic regions^25^—such as northern Portugal, Sweden, Japan and Brazil—harbour founder mutations like V30M, with prevalence rates reaching over 1600 per million in some areas.^25^ In contrast, the Val122Ile variant, associated with cardiac involvement, is present in ~3%–4% of African-Americans in the USA, though penetrance is incomplete.^26^

ATTRwt occurs sporadically and primarily affects older men. Autopsy studies reveal that over 25% of men above age 80 years have cardiac ATTR deposits, most of which go unrecognised during life.^21^ Prospective imaging studies suggest that up to 13% of patients with heart failure with preserved ejection fraction (HFpEF) and 12% of those undergoing transcatheter aortic valve replacement have cardiac ATTR. These findings highlight the extent of underdiagnosis in elderly populations with cardiac symptoms.^27^

ATTR imposes substantial morbidity and mortality. ATTRv may present in mid-life with progressive peripheral neuropathy, autonomic dysfunction and/or CM.^28^ Without treatment, polyneuropathy leads to severe disability within a decade, while CM results in heart failure and shortened survival.^29^ ATTRwt typically manifests after age 65–70 years as slowly progressive heart failure, often preceded by carpal tunnel syndrome or spinal stenosis.^30^ Diagnostic delay is common, especially in ATTRwt, where symptoms are often misattributed to age-related heart disease.

Survival in untreated cardiac ATTR ranges from 2 years to 5 years, comparable to advanced heart failure. Recent studies report 2-year mortality rates between 10% and 30% for ATTRwt-CM and between 10% and 50% for ATTRv cases.^31^ The disease burden includes heart failure hospitalisations, arrhythmias, pacemaker implantation, stroke and neuropathic complications—factors that significantly impair QOL.^31^

With the emergence of disease-modifying therapies such as TTR stabilisers and gene silencers, early diagnosis is essential. Systematic screening has been shown to increase recognition of ATTR-CM, underscoring the need for heightened clinical suspicion in at-risk populations. Despite its historical classification as rare, ATTR is now recognised as a clinically significant cause of heart failure and neuropathy in elderly individuals and mutation carriers.

## Current diagnostic approaches in ATTR

Diagnosis of ATTR requires confirmation of amyloid deposits and identification of TTR as the precursor protein. Distinction from light-chain (AL) amyloidosis is critical, as management differs substantially. The diagnostic strategy incorporates clinical assessment, genetic testing, imaging, biopsy and laboratory evaluation.

### Genetic testing

In cases of pathologically confirmed or strongly suspected ATTR, genetic testing of the *TTR* gene is essential to differentiate hereditary (ATTRv) from wild type (ATTRwt) forms. *TTR* gene sequencing via blood DNA analysis detects pathogenic variants, with significant clinical and familial implications. A positive result confirms ATTRv, warranting cascade testing and genetic counselling for at-risk relatives. Conversely, a negative result in confirmed ATTR indicates ATTRwt. Genetic testing is recommended for all patients with ATTR, including elderly individuals with typical ATTRwt phenotypes, as late-onset pathogenic variants may occasionally be identified.

### Biopsy and histopathology

Tissue biopsy remains the diagnostic gold standard for amyloidosis, particularly when non-invasive tests are inconclusive or clinical features are atypical. Amyloid can be sampled from affected organs or surrogate sites such as subcutaneous fat.

#### Abdominal fat pad biopsy

This minimally invasive procedure detects amyloid via Congo red staining.^32 33^ Sensitivity is high in AL amyloidosis (~80%–85%)^34^ and in ATTRv (~90%).^35^ However, a negative result does not exclude ATTR, particularly wild type. Positive samples enable amyloid typing through immunohistochemistry or mass spectrometry.

#### Endomyocardial biopsy

Endomyocardial biopsy (EMB) offers definitive diagnosis of cardiac amyloidosis. Congo red staining^32 33^ with apple-green birefringence confirms amyloid, with TTR typing achieved via immunohistochemistry or mass spectrometry. Though invasive, EMB is safe in experienced centres and is crucial when non-invasive tests are inconclusive or AL amyloidosis remains a concern.

#### Other tissue biopsies

In select cases, biopsies from peripheral nerve, gastrointestinal tract, kidney, carpal ligament or vitreous fluid may aid diagnosis. These are generally reserved for complex presentations.

### Imaging techniques

Modern imaging has enhanced the detection of cardiac ATTR and guides further diagnostic evaluation.

#### Echocardiography

Common initial modality. Suggestive features include increased left ventricular (LV) wall thickness (≥12 mm), preserved ejection fraction with diastolic dysfunction, biatrial enlargement and valvular thickening.^36^ Longitudinal strain imaging often reveals apical sparing, a distinguishing marker. While indicative, echocardiography cannot differentiate ATTR from AL amyloidosis.

#### Bone scintigraphy

Technetium-labelled tracers (99m pyrophosphate; 99m PYP), 3,3-diphosphono-1,2-propanodicarboxylic acid (DPD), hydroxymethylene diphosphonate (HMDP) bind myocardial ATTR deposits. Cardiac uptake ≥grade 2 (equal to or greater than bone) is highly specific for ATTR in the absence of monoclonal proteins, allowing non-biopsy diagnosis.^37^ Biopsy is required in cases with monoclonal gammopathy or equivocal uptake.

#### Cardiac magnetic resonance

Cardiac magnetic resonance (CMR) with gadolinium identifies amyloid via diffuse subendocardial or transmural Late Gadolinium Enhancement (LGE) and elevated native T1/ECV.^38^ While sensitive and specific (~85%–90%), CMR cannot distinguish ATTR from AL. It is valuable when echocardiographic findings are inconclusive and for prognostication.

#### Other imaging

Electrocardiogram (ECG) may show low voltage despite left ventricular (LV) hypertrophy.^39^ Musculoskeletal signs (eg, bilateral carpal tunnel syndrome, tendon rupture) may suggest ATTRwt.^40^ In neuropathic ATTRv, nerve conduction and autonomic tests^41^ support but do not confirm diagnosis, which requires genetic testing and/or biopsy.

### Laboratory tests and biomarkers

Although no circulating biomarker definitively diagnoses ATTR, several laboratory assessments are critical in the diagnostic algorithm. A monoclonal protein screen—including serum and urine electrophoresis and serum-free light AL—is essential to exclude AL amyloidosis, which necessitates distinct treatment. Cardiac biomarkers such as N-terminal pro-B-type natriuretic peptide (NT-proBNP) and troponin are typically elevated in ATTR-CM^42^ and serve as indicators of myocardial involvement and prognostic tools, though they lack disease specificity.

In the context of ATTR neuropathy, neurofilament light chain (NfL) has emerged as a promising biomarker of axonal injury. Elevated serum or plasma NfL levels have been correlated with disease severity and progression in ATTRv polyneuropathy,^43^ and reductions in NfL levels have been observed in patients responding to treatment. While still under investigation, NfL may eventually support earlier diagnosis and therapeutic monitoring.

## Liver transplantation for ATTRv

The first successful treatment of ATTRv was liver transplantation, which was introduced in 1990.^44^ The rationale behind the treatment was to exchange the patient’s amyloidogenic variant TTR synthesising liver with one that only produced wild type TTR and thereby halting amyloid formation. Even though the procedure in many cases was able to halt the disease progression, it became obvious that the disease continued to progress in many cases, and that this was caused by continued amyloid formation from wild type TTR. Therefore, successful treatment needed to target both wild type and variant TTR. Today, liver transplantation as a treatment for ATTRv has been abandoned following the introduction of effective pharmacological alternatives.

## Pharmacological approaches targeting TTR

**Figure 1:**
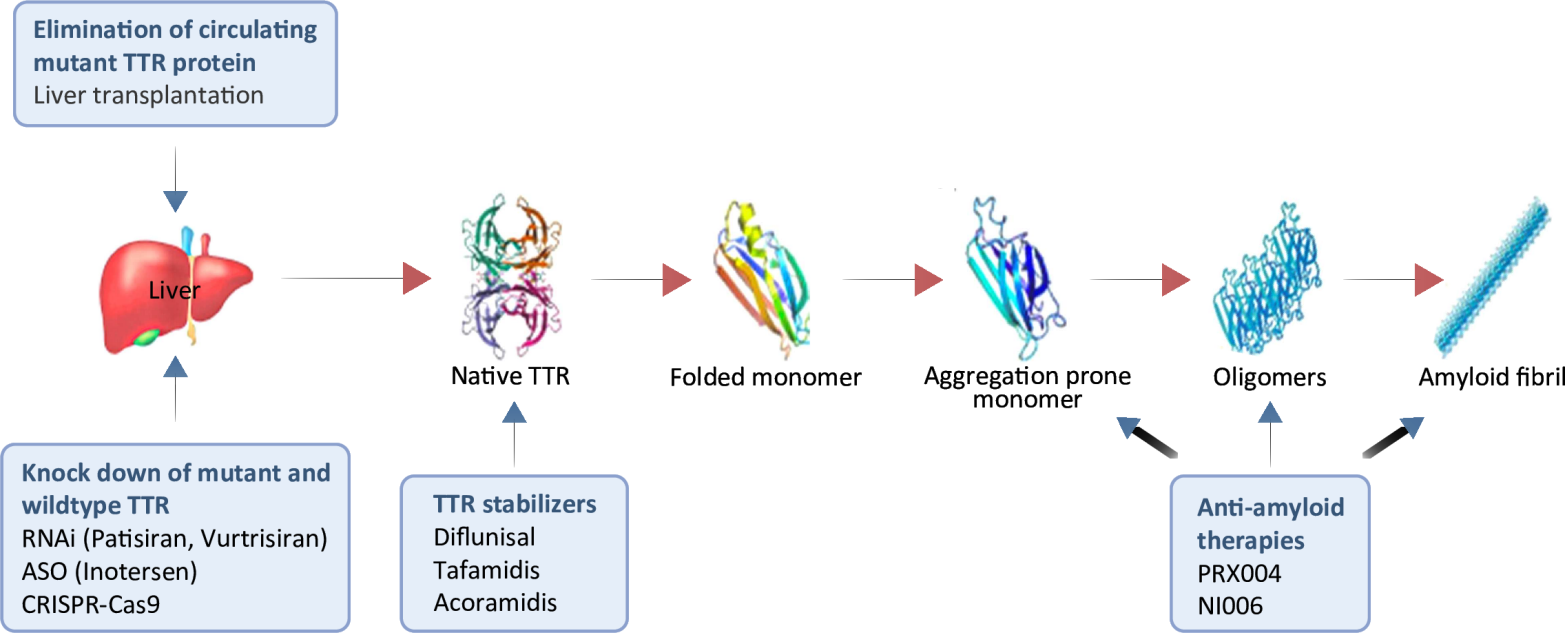
Pharmacotherapeutic strategies targeting the fundamental pathobiology of ATTR. It elucidates the series of events involving the dissociation, misfolding and aggregation of TTR proteins, resulting in the formation of amyloid fibrils. It also higlights various therapeutic approaches and existing pharmacological treatments for the management of ATTRv. ASO, antisense oligonucleotide; ATTR, transthyretin amyloidosis; ATTRv, hereditary ATTR; CRISPR, clustered regularly interspaced short palindromic repeats; RNAi, RNA interference; TTR, transthyretin.

Novel and emerging therapies specifically targeting TTR may be categorised into three distinct groups based on their pharmacological mechanisms to counteract the effects of TTR disassembly and amyloid formation (figure 1 and table 1): (1) TTR stabilisers; (2) *TTR* gene silencers or knockdown; and (3) Removal of misfolded TTR and ATTR.

**Table 1:** Novel and evolving pharmacological treatments for ATTRv: interventions involving the stabilisation and silencing of TTR, as well as agents targeting amyloid deposition

Drugs	Mechanism	Administration	Status	Drug provider
Diflunisal	TTR stabiliser	Oral	Approved as NSAID, off-label use, ATTRv with PN or CM and ATTRwt	Generic (various manufacturers)
Tafamidis	TTR stabiliser	Oral	Approved for ATTRwt and ATTRv-CM with/without stage I PN and stage I ATTRv-PN	Pfizer
Acoramidis	TTR stabiliser	Oral	Approved for ATTR-CM	BridgeBio/Eidos Therapeutics
Inotersen	ASO inhibitor of TTR production	284 mg subcutaneous, once a week	Approved for ATTRv-PN stage I or II	Ionis Pharmaceuticals
Patisiran	siRNA targeting TTR mRNA	0.3 mg/kg, intravenous every 3 weeks	Approved for ATTRv-PN stage I or II	Alnylam Pharmaceuticals
Vutrisiran	siRNA targeting TTR mRNA	25 mg subcutaneous, every 3 months	Approved for ATTRv-PN stage I or II	Alnylam Pharmaceuticals
Epleontersen	ASO inhibitor of TTR production	Subcutaneous every 4 weeks	Approved for ATTRv-PN	Ionis Pharmaceuticals/AstraZeneca
NTLA-2001	CRISPR/Cas9 editing of TTR	One-time infusion	Phase III clinical trial	Intellia Therapeutics/Regeneron
PRX004	mAb designed to bind and remove ATTR deposits	Intravenous every 28 days	Phase I	Novo Nordisk (acquired from Prothena)
NI006	Recombinant IgG1 human mAb directed against TTR	Intravenous infusion every 4 months	Phase I	Neurimmune/AstraZeneca

ASO, antisense oligonucleotide; ATTR, transthyretin amyloidosis; ATTR-CM, ATTR with cardiomyopathy; ATTRv, hereditary ATTR; ATTRv-CM, ATTRv with cardiomyopathy; ATTRv-PN, ATTRv with polyneuropathy; ATTRwt, wild type ATTR; CM, cardiomyopathy; CRISPR, clustered regularly interspaced short palindromic repeats; mAb, monoclonal antibody; mRNA, microRNA; NSAID, non-steroidal anti-inflammatory drug; PN, polyneuropathy; siRNA, small interfering RNA; TTR, transthyretin.

1. TTR stabilisers are pharmacological agents designed to enhance the structural integrity and stability of TTR. These agents function by binding specifically to TTR tetramers, thereby reducing the propensity of TTR to dissociate into monomers, which is a critical step in the pathogenesis of TTR amyloidosis.^45 46^ By stabilising TTR in its native tetrameric form, these stabilisers mitigate the accumulation of misfolded TTR protein and subsequent amyloid deposition in various tissues.
2. Gene silencing approaches aim to mitigate TTR protein expression at the transcriptional or post-transcriptional level, thereby attenuating amyloid deposition and halting disease progression. One prominent modality for *TTR* gene silencing involves the utilisation of small nucleic acid-based molecules, including small interfering RNAs (siRNAs) and antisense oligonucleotides (ASOs).
  siRNAs are double-stranded RNA molecules typically 20–25 nucleotides in length that function within the RNA interference (RNAi) pathway.^47^ They exert their gene-silencing effects by guiding the RNA-induced silencing complex to complementary target mRNA molecules encoding TTR. On binding, siRNAs facilitate the cleavage and subsequent degradation of target mRNA, thereby reducing TTR protein synthesis.
  Similarly, ASOs are synthetic single-stranded nucleic acid molecules, typically 15–25 nucleotides in length, designed to hybridise with specific target mRNA sequences through Watson-Crick base pairing.^48^ This binding event can trigger mRNA degradation via RNase H-mediated cleavage or interfere with mRNA translation by steric hindrance, ultimately leading to diminished TTR protein synthesis.
3. The third therapeutic modality for addressing TTR amyloidosis involves antiamyloid antibodies,^49 50^ which are specifically designed to bind to and extract misfolded TTR and amyloid deposits from affected organs and tissues. These treatments are of paramount importance, since patients diagnosed with advanced-stage disease, characterised by pronounced amyloid deposition and organ dysfunction, such as pronounced heart failure, currently are without effective treatment options.

## Current treatment approaches

### TTR stabilisers

#### Tafamidis

Tafamidis was the first pharmacological agent that underwent a clinical trial for the treatment of ATTRv with polyneuropathy (ATTRv-PN). It exerts its therapeutic action by binding to the T4-binding site of TTR, thereby facilitating stabilisation of the TTR tetramer. Notably, in a pivotal phase III clinical trial conducted in 2012 its efficacy in patients with ATTRv-PN caused by the V30M *TTR* gene mutation failed to meet its primary predetermined clinical endpoint.^51^ However, 21% of the participants underwent liver transplantation, and since they per protocol were classified as ‘nonresponders’, the statistical power of the trial was diminished. Thus, in the intention-to-treat analysis, the treatment arm of the study fell marginally short of reaching statistical significance (p=0.068),^51^ but a comprehensive scrutiny of the data revealed compelling evidence for tafamidis’ decelerating effect on the progression of the patients’ peripheral neurological impairment. Tafamidis was approved by several regulatory authorities worldwide including the EU for ATTRv-PN, but it notably did not receive approval from the US Food and Drug Administration (FDA).^51^

In 2018, a randomised controlled clinical trial titled ATTRACT (Tafamidis in Transthyretin Amyloid Cardiomyopathy) found that tafamidis in patients with ATTR-CM and New York Heart Association functional class I–III symptoms benefited from tafamidis treatment.^52^ This pivotal trial encompassed a cohort of 441 patients diagnosed with ATTR-CM, who were randomly allocated in a double-blind fashion, using a 2:1:2 ratio, to receive either tafamidis 80 mg, tafamidis 20 mg or placebo once a day over a period of 30 months. Tafamidis treatment significantly reduced the incidence of all-cause mortality in comparison to the placebo group, as well as a diminished rate of hospitalisations attributable to cardiovascular causes. Additionally, tafamidis treatment had a mitigating effect on the decline in functional capacity and QOL at the conclusion of the 30-month observation period, evaluated by the 6 min walk distance and scores on the Kansas City Cardiomyopathy Questionnaire-Overall Summary. Subsequent analysis revealed that the significant reductions in mortality and functional decline with tafamidis treatment were consistent across both wild type and variant subtypes.^53^ The occurrence of adverse events was comparable between the tafamidis and placebo groups. Tafamidis has been approved for the treatment of ATTRwt-CM and ATTRv-CM by the FDA and the European Medicines Agency (EMA).

#### Diflunisal

Diflunisal, a readily available and economically feasible non-steroidal anti-inflammatory drug, possesses potent TTR-stabilising capacity and has been used in the management of ATTR. It stabilises the TTR tetramer through specific binding to the T4-binding site. A randomised, double-blind, placebo-controlled trial published in 2013 with 130 patients with ATTRv-PN showed that diflunisal at a dose of 250 mg two times per day administered over a period of 2 years significantly diminished neurological impairment progression and preserved the patients’ QOL.^54^ Another open-label study, comprising 54 patients diagnosed with ATTRv with a variant, conducted over a duration of 24 months and administered with diflunisal, yielded findings indicating the maintenance of stable neurological function and nutritional status among the patient cohort.^55^ In the context of managing ATTR-CM, one retrospective study comprising 81 participants (41% receiving diflunisal) revealed beneficial variances in Left Atrial Volume Index and troponin I levels on follow-up assessment (median interval 1 year) among those subjected to diflunisal therapy.^56^ Another retrospective analysis involving 104 subjects (35 of whom received diflunisal) established a correlation between diflunisal administration and enhanced survival rates following adjustments for pertinent covariates such as age, baseline brain natriuretic peptide, estimated glomerular filtration rate, troponin I, interventricular septal thickness and LV ejection fraction.^57^

## Gene silencers

### Patisiran

Patisiran represents a pioneering RNAi therapeutic agent encapsulated within lipid nanoparticles, facilitating its uptake into hepatocytes for the specific targeting of TTR protein. In the 2018 APOLLO Trial encompassing patients diagnosed with ATTRv-PN (n=225, PND Score I–IIIb), a randomised, double-blind protocol with a 2:1 allocation ratio to either patisiran or placebo was employed.^58^ This pivotal phase III investigation revealed that patisiran elicited a substantial reduction in polyneuropathy-related symptoms over an 18-month period, as evidenced by the modified Neuropathy Impairment Score+7 (mNIS+7) from baseline. Furthermore, patisiran therapy exhibited favourable outcomes in terms of enhancing QOL and ameliorating autonomic dysfunction, encompassing orthostatic intolerance, gastrointestinal manifestations and nutritional status.^59^ Subsequent to the compelling efficacy demonstrated in the APOLLO Trial, patisiran received regulatory approval from the EMA and the FDA for the treatment of ATTRv-PN. Additionally, emerging evidence suggests the therapeutic benefits of patisiran in individuals afflicted with ATTR-CM. A subgroup analysis derived from the APOLLO investigation underscored notable improvements associated with patisiran therapy, including reductions in mean LV wall thickness, enhancements in global longitudinal strain (GLS), lowered levels of NT-proBNP, and a diminished incidence of adverse cardiac events compared with placebo following an 18-month intervention period.^60^

Long-term data from a 5-year open-label extension of the APOLLO Study further reinforced the durable efficacy and safety of patisiran in patients with ATTRv-PN. Patients treated continuously with patisiran maintained or improved their neurological function and QOL over the extended treatment period, with a favourable safety profile and no new safety signals observed.^61^

### Vutrisiran

Vutrisiran represents a next-generation RNAi therapeutic compound that is conjugated with N-acetyl galactosamine (GalNAc). GalNAc exhibits a pronounced affinity for the asialoglycoprotein receptor, abundantly expressed in hepatocytes, thereby facilitating targeted delivery of the drug to the liver. Unlike its predecessor, patisiran, vutrisiran is administered subcutaneously every 3 months, obviating the need for the intravenous infusions required by patisiran, which are administered every 3 weeks. Notably, vutrisiran administration does not necessitate premedication to mitigate infusion-related reactions, attributed to its formulation devoid of lipid nanoparticles. The HELIOS-A Trial, comprising 164 participants, investigated the efficacy and safety of vutrisiran compared with an external placebo group derived from the APOLLO Trial.^62^ Patients diagnosed with ATTRv-PN were randomly assigned in a 3:1 ratio to receive either subcutaneous vutrisiran every 3 months or intravenous patisiran every 3 weeks over an 18-month period. In addition to achieving all predetermined secondary endpoints, vutrisiran treatment yielded a statistically significant enhancement in the mNIS+7 group compared with the placebo group (p=3.54 × 10^–12^). Based on these compelling findings, vutrisiran has received approval from the EMA and the FDA for the management of ATTRv-PN (PND Score I–IIIb). The HELIOS-B Trial is an ongoing phase III trial to evaluate the therapeutic efficacy of vutrisiran in patients diagnosed with ATTR-CM (ClinicalTrials.gov Identifier: NCT04153149). The primary endpoint of HELIOS-B encompasses the composite assessment of all-cause mortality and recurrent cardiovascular events, including cardiovascular hospitalisations and urgent visits due to heart failure.

In March 2025, the FDA approved an expanded indication for vutrisiran to include the treatment of ATTR-CM, based on interim findings from the HELIOS-B Trial demonstrating reductions in cardiovascular-related hospitalisations and all-cause mortality.^63^

### Inotersen

Inotersen, an ASO, functions by impeding hepatic synthesis of TTR. The NeuroTTR Trial provided evidence of inotersen’s beneficial impact on the progression of neurological disease and enhancement of QOL.^64^ Nonetheless, notable adverse events were recorded, including instances of glomerulonephritis (3%) and severe thrombocytopenia (3%, defined as a platelet count <25 × 10^3^ /µL). It is noteworthy that within the inotersen-treated group, five fatalities occurred, one of which was attributable to fatal intracranial haemorrhage associated with severe thrombocytopenia. Conversely, no fatalities were reported in the control group. Subsequent to the NeuroTTR Trial, inotersen received approval from EMA and FDA for the treatment of variant ATTRv-PN. However, given the emergence of extended-release ASOs exhibiting more favourable safety profiles, inotersen is no longer undergoing evaluation for ATTR-CM.

### Eplontersen

Eplontersen is an ASO that shares an identical nucleotide sequence with inotersen. Unlike inotersen, eplontersen is chemically linked to a triantennary N-acetylgalactosamine moiety, enhancing receptor-mediated uptake by hepatocytes. This modification amplifies the potency of the drug, facilitating lower and less frequent dosing regimens.^65^ CardioTTRansform (ClinicalTrials.gov Identifier: NCT04136171) and NeuroTTRansform (ClinicalTrials.gov Identifier: NCT04136184) represent phase III clinical trials investigating the efficacy and safety of eplontersen in patients diagnosed with ATTR-CM and ATTRv-PN, respectively. In the NeuroTTRansform Trial, a comprehensive efficacy evaluation at week 66 will juxtapose the outcomes of the eplontersen treatment arm with an external placebo arm derived from the NEURO-TTR Trial. Additionally, a predefined interim analysis conducted at week 35 disclosed that eplontersen elicited an 81.2% mean reduction in the co-primary endpoint of serum TTR concentration compared with baseline (p<0.001).^66^ Relative to the external placebo cohort, patients receiving eplontersen manifested decelerated progression of neuropathic disease (p<0.001) and reported enhancements in QOL (p<0.001). In contrast to its precursor, inotersen, eplontersen exhibited a favourable safety and tolerability profile, including stable platelet values. Eplontersen, marketed as Wainua, has received regulatory approval from both the FDA and EMA for the treatment of adult patients with ATTRv-PN. These approvals were granted based on interim findings from the phase III Neuro-TTRansform Trial,^66^ which demonstrated clinically meaningful improvements in neuropathy impairment and patient-reported QOL. Ongoing data from the CardioTTRansform Trial are expected to provide further insights into the efficacy of eplontersen in patients with ATTR-CM.

### Acoramidis

Acoramidis, previously designated as AG10, represents another promising therapeutic agent in the pipeline for TTR stabilisation, featuring a distinct mechanism of action. An antecedent investigation uncovered a remarkably stabilising mutation within TTR (T119M), which precipitated a substantial reduction in the dissociation rate of TTR tetramers, exceeding 33-fold compared with the wild type counterpart.^45^ This T119 variant mediates TTR tetramer stabilisation through the facilitation of hydrogen bond formation between neighbouring serine residues situated at position 117 of each monomeric unit.^67^ Acoramidis, engineered as a potent and highly selective oral TTR stabiliser, is intricately designed to emulate the structural effects conferred by this protective mutation.^68^ Preliminary findings from a phase II clinical investigation revealed favourable tolerability profiles associated with acoramidis, along with the demonstration of near-complete stabilisation of TTR tetramers and normalisation of low serum TTR levels.

The ATTRibute-CM Trial (ClinicalTrials.gov Identifier: NCT03860935) was a global, phase III, randomised, double-blind, and placebo-controlled study designed to assess the efficacy and safety of acoramidis in patients with ATTR-CM. Although part A of the trial, which evaluated change in the 6 min walk distance at 12 months, did not achieve statistical significance, the final analysis from part B demonstrated a significant clinical benefit of acoramidis compared with placebo.^69^ Specifically, acoramidis showed superiority on a hierarchical composite endpoint that included all-cause mortality, frequency of cardiovascular-related hospitalisations, 6 min walk test performance and health-related QOL over a 30-month period. These benefits were observed across both hereditary (ATTRv) and wild type (ATTRwt) forms of the disease. Based on these findings, acoramidis received regulatory approval from both the FDA and EMA for the treatment of ATTR-CM.

## Emerging therapies

### CRISPR-Cas9 gene editing

Recent advances in gene technology have made it possible to manipulate the *TTR* gene within living organisms, using the clustered regularly interspaced short palindromic repeats (CRISPR) and associated Cas9 endonuclease (CRISPR-Cas9) system.^70^ Initially identified as part of bacterial immune defences against viral invasions, CRISPR-Cas9 has rapidly gained prominence as a versatile tool in laboratories globally. This prominence is attributable to its capacity to induce targeted DNA double-strand breaks at specific loci within intricate endogenous genomes, facilitated by short guide RNA sequences.^71 72^ These induced breaks enable precise gene editing mechanisms, including homology-directed repair, or alternately, perturbation of gene function through insertion-deletion mutations introduced via non-homologous end-joining mechanisms. The recognition of the potential of this technology has led researchers to acknowledge its significant promise in mitigating monogenic human disorders through singular in vivo gene therapy administration.^73 74^

Numerous attributes of the *TTR* gene render it an advantageous target for the CRISPR-Cas9 system. Given that over 95% of TTR is synthesised in the liver and its principal functions involve the transportation of retinol-binding protein and T4, selective knockout of this gene can be achieved with minimal systemic repercussions, as it predominantly impacts a single cell type: the hepatocyte.^75 76^ The primary aim of a CRISPR-Cas9-based strategy is to obviate the necessity for recurrent, long-term administrations to sustain *TTR* suppression.^77^ Consequently, researchers have developed NTLA-2001, a CRISPR-Cas9-based platform designed to induce *TTR* knockout, which is delivered via lipid nanoparticles specifically targeting hepatocytes expressing apolipoprotein E.^70 77^ Preclinical evaluations in animal models substantiated the efficacy of NTLA-2001.^78^

In an ongoing phase I clinical trial, NTLA-2001 has demonstrated substantial, dose-dependent reductions in serum TTR concentrations following a single intravenous infusion.^79^ Notably, these reductions have been deep and durable, with follow-up extending beyond 2 years in certain cohorts. The treatment has been generally well tolerated, with most adverse events reported as mild. Importantly, there has been no evidence of off-target genomic alterations or oncogenic transformations. As of 2024, the trial continues with cohort expansion to further assess safety and efficacy across a broader patient population. These findings exemplify a groundbreaking proof of concept for the application of CRISPR-Cas9 in medical therapeutics, with potential far-reaching implications for the treatment of various diseases through precise gene-editing methodologies.

### Antiamyloid therapies

There are concerted efforts to harness the potential of monoclonal antibodies (mAbs) for the degradation of amyloid deposits. PRX004, a humanised mAb, is engineered to target pathogenic variants of TTR by recognising an epitope located within positions 89–97, which is exposed in non-native conformations or disaggregated monomers of the protein but concealed within the normal TTR tetramer.^80^,^82^ A phase I clinical trial investigating PRX004 enrolled 21 patients diagnosed with ATTRv. Patients were excluded if they had planned liver transplantation or recent exposure to patisiran or inotersen, although concurrent treatment with tafamidis or diflunisal was permissible. Due to the COVID-19 epidemic, the study was terminated prematurely. However, favourable changes in cardiac function, as evaluated by LV GLS, were observed in all seven evaluable patients treated with PRX004, exhibiting a mean change in GLS of −1.21% at 9 months.^83^ Furthermore, these patients demonstrated slower progression of neuropathic disease at 9 months, evidenced by a mean change in neuropathy impairment score of +1.29 points, which is more favourable than the expected natural progression of +9.2 points.^84^ PRX004 was found to be safe and well tolerated across all six doses tested. Following these encouraging preliminary findings, Novo Nordisk acquired PRX004 from Prothena in July 2021 and re-designated it as NNC6019. A phase II randomised controlled trial (NCT05442047) is currently underway to further evaluate the safety and efficacy of NNC6019 in patients with ATTR-CM.^85^

Moreover, through a high-throughput screen of human memory B cell libraries, investigators have identified a repertoire of ATTR-binding antibodies characterised by high-affinity binding to ATTR, robust amyloid removal activity and negligible binding to normal TTR.^81^ Among these, NI301A, an mAb, specifically recognises the linear epitope WEPFA within positions 41–45 of TTR, which remains concealed within the native conformation of the protein but becomes accessible following unfolding and aggregation of ATTR deposits. Preclinical studies have demonstrated NI301A’s capacity to induce amyloid clearance by macrophages via Fc-mediated phagocytosis. NI301A underwent evaluation in a phase I clinical trial (NCT04360434) under the designation NI006 including 40 patients with ATTRv-CM or ATTRwt-CM. The study showed that over a span of 12 months, there was an observed decline in the cardiac amyloid burden associated with amyloidosis.^86^ Coinciding with this trend, there was a notable decrease noted in the median concentrations of N-terminal pro-B-type natriuretic peptide and troponin T.^86^

## Conclusions and perspectives

In recent years, substantial progress has been achieved in the treatment of ATTR, fundamentally transforming the clinical outlook for affected individuals. The introduction of TTR stabilisers, gene-silencing therapies, and emerging disease-modifying approaches—including mAbs and CRISPR-based genome editing—has enabled a more comprehensive and multifaceted approach to disease management. These therapeutic advancements, in combination with innovations in non-invasive diagnostic techniques such as scintigraphy and advanced cardiac imaging, have significantly improved the potential for early detection, which is crucial for optimising treatment outcomes.

Despite these remarkable developments, several important challenges and unanswered questions persist. One major gap is the absence of head-to-head clinical trials comparing the efficacy and safety of different therapeutic classes. Such comparative studies are essential to inform personalised treatment strategies and clinical decision-making. Additionally, while multiple treatment modalities are now available or in late-stage development, the optimal sequencing of therapies—or whether combination treatments may confer additive or synergistic benefits—remains unclear and warrants systematic evaluation.

Another concern is the high cost and variable accessibility of novel therapies, particularly gene-based treatments and biologics. There is a pressing need for real-world data on cost-effectiveness and long-term clinical benefit, as well as strategies to ensure equitable access across diverse healthcare settings. Furthermore, patients diagnosed at advanced stages of cardiac or neurological involvement continue to face poor prognoses, underscoring the urgency of developing interventions capable of reversing established organ damage or removing existing amyloid deposits.

The long-term safety and durability of gene-silencing and gene-editing therapies also remain areas of active investigation. As these technologies are increasingly integrated into clinical practice, ongoing surveillance will be critical to detect potential off-target effects and assess sustained treatment efficacy over time. In parallel, the expanding use of genetic screening is identifying more individuals who carry pathogenic TTR mutations but have not yet developed symptoms. Guidelines are still lacking on when to initiate therapy in such presymptomatic carriers to delay or prevent disease onset.

In terms of disease monitoring, promising biomarkers such as NfL, NT-proBNP and advanced imaging parameters are under investigation, but further validation and standardisation are needed before they can be widely adopted in clinical practice. Looking forward, the integration of personalised medicine, genomic technologies and longitudinal patient registries will be central to advancing care for ATTR. Multidisciplinary collaboration—among neurologists, cardiologists, geneticists and pharmacologists—will play a pivotal role in refining therapeutic strategies and delivering holistic, patient-centred care. Although ATTR remains a complex and challenging disorder, the expanding pipeline of emerging therapies, combined with an increasingly sophisticated understanding of disease biology, offers renewed hope for delaying disease onset, halting progression, and ultimately preserving organ function and QOL.
