# Supplementary material for: Advances in the treatment of transthyretin amyloidosis
Source: eGastroenterology. 2025 Jul 18;3(3):e100198. doi: 10.1136/egastro-2025-100198 (PMC12278169; doi:10.1136/egastro-2025-100198)
Supplement: Supplementary file 1 [file egastro-2025-100198.reviewer_comments.pdf]

## PEER REVIEW HISTORY

eGastroenterology publishes all reviews undertaken for accepted manuscripts. Reviewers are asked to complete a checklist review form and are provided with free text boxes to elaborate on their assessment. These free text comments are reproduced below.

## ARTICLE DETAILS

| | |
|---|---|
| **TITLE (PROVISIONAL)** | Advances in the Treatment of Transthyretin Amyloidosis |
| **AUTHOR** | Intissar Anan |

## VERSION 1 - REVIEW

| | |
|---|---|
| **REVIEWER NAME** | Zhang, Kezhong |
| **REVIEWER AFFILIATION** | Wayne State University |
| **REVIEWER CONFLICT OF INTEREST** | No |
| **AI DISCLOSURE** | No, I have not used AI |
| **DATE REVIEW RETURNED** | 09-Mar-2025 |

| | |
|---|---|
| **GENERAL COMMENTS** | In this manuscript, the author did a nice job in summarizing the advances in the treatment of Transthyretin Amyloidosis. In particular, the manuscript provides a comprehensive overview of the current and emerging approaches to treat Transthyretin Amyloidosis. The interpretations and discussions are concise and easy to understand. It will be an informative and interesting piece to the Transthyretin Amyloidosis research community. The major comments include:<br>1) The literature was cited until 2023. This needs to be updated. In 2024 alone, there were over 80 publications addressing the topic of treatment of Transthyretin Amyloidosis.<br>2) The introduction section needs to be improved. Descriptions of the mechanism and pathogenesis of Transthyretin Amyloidosis should be included or expanded.<br>3) For the last section, a perspective of the future direction in treating Transthyretin Amyloidosis should be included. Particularly, the challenges and the major questions to be resolved in the field should be highlighted in a section “Conclusion and Perspective”. |

| | |
|---|---|
| **REVIEWER NAME** | Yang, Ling |
| **REVIEWER AFFILIATION** | The University of Iowa Roy J and Lucille A Carver College of Medicine, Department of Anatomy and Cell Biology |
| **REVIEWER CONFLICT OF INTEREST** | No |
| **AI DISCLOSURE** | No, I have not used AI |
| **DATE REVIEW RETURNED** | 21-Mar-2025 |

| | |
|---|---|
| **GENERAL COMMENTS** | In this manuscript, the author summarizes and discusses existing therapies and therapeutic candidates for transthyretin amyloid-related amyloidosis (ATTR). This review provides valuable insights into the understudied field of ATTR. Following are suggestions for the manuscript: |

| | |
|---|---|
| | 1. The manuscript would benefit from including information regarding the epidemiology and disease burden of ATTR.<br>2. It would be helpful to provide brief background on the biology and nature of transthyretin (TTR). For instance, clearly state that hepatocytes are the primary cells responsible for producing TTR and discuss the number of inherited genetic variants of TTR.<br>3. To improve the coherence of the introduction section, this reviewer suggests reorganizing the descriptions of ATTRv (variant ATTR) and ATTRwt (wild-type ATTR) into distinct subsections.<br>4. Please include a brief summary of the current diagnostic methods for ATTR.<br>5. Perspectives could be included in the Conclusions section to discuss remaining questions in the field and the future research/therapy directions.<br>6. Please include the information about Drugs providers in the table 1. |

**VERSION 1 – AUTHOR RESPONSE**

Reviewer #1:

Comments to the Author

In this manuscript, the author did a nice job in summarizing the advances in the treatment of Transthyretin Amyloidosis. In particular, the manuscript provides a comprehensive overview of the current and emerging approaches to treat Transthyretin Amyloidosis. The interpretations and discussions are concise and easy to understand. It will be an informative and interesting piece to the Transthyretin Amyloidosis research community. The major comments include:

1) The literature was cited until 2023. This needs to be updated. In 2024 alone, there were over 80 publications addressing the topic of treatment of Transthyretin Amyloidosis.

Response: Thank you for pointing this out. The literature review has been updated to include recent publications from 2024 and 2025. Additional references have been incorporated to reflect the latest advancements in the treatment of Transthyretin Amyloidosis.

2) The introduction section needs to be improved. Descriptions of the mechanism and pathogenesis of Transthyretin Amyloidosis should be included or expanded.

Response: I appreciate your suggestion. The introduction has been expanded to provide a more detailed discussion of the mechanism and pathogenesis of Transthyretin Amyloidosis. The revised version includes an in-depth explanation of how transthyretin misfolding leads to amyloid deposition and organ dysfunction.

3) For the last section, a perspective of the future direction in treating Transthyretin Amyloidosis should be included. Particularly, the challenges and the major questions to be resolved in the field should be highlighted in a section “Conclusion and Perspective”.

Response: A new section titled "Conclusion and Perspective" has been added to discuss future directions in treating Transthyretin Amyloidosis.

Reviewer #2: Comments to the Author

In this manuscript, the author summarizes and discusses existing therapies and therapeutic candidates for transthyretin amyloid-related amyloidosis (ATTR). This review provides valuable insights into the understudied field of ATTR. Following are suggestions for the manuscript:

1. The manuscript would benefit from including information regarding the epidemiology and disease burden of ATTR.

Response: Thank you for your comment. A section summarizing the epidemiology and disease burden of ATTR has been added.

2. It would be helpful to provide brief background on the biology and nature of transthyretin (TTR). For instance, clearly state that hepatocytes are the primary cells responsible for producing TTR and discuss the number of inherited genetic variants of TTR.

Response: A brief explanation of transthyretin biology has been added.

3. To improve the coherence of the introduction section, this reviewer suggests reorganizing the descriptions of ATTRv (variant ATTR) and ATTRwt (wild-type ATTR) into distinct subsections.

Response: The introduction section has been reorganized into distinct subsections for ATTRv (variant ATTR) and ATTRwt (wild-type ATTR) to improve clarity and coherence.

4. Please include a brief summary of the current diagnostic methods for ATTR.

Response: A summary of current diagnostic methods for ATTR has been added.

5. Perspectives could be included in the Conclusions section to discuss remaining questions in the field and the future research/therapy directions.

Response: In response to your suggestion, perspectives on future research directions and unresolved questions in the field have been incorporated into the revised conclusion section.

6. Please include the information about Drugs providers in the table 1.

Response: Information about drug providers has been included in Table 1.

**VERSION 2 - REVIEW**

| | |
|---|---|
| **REVIEWER NAME** | Zhang, Kezhong |
| **REVIEWER AFFILIATION** | Wayne State University |
| **REVIEWER CONFLICT OF INTEREST** | No |
| **AI DISCLOSURE** | No, I have not used AI |
| **DATE REVIEW RETURNED** | 10-May-2025 |

| | |
|---|---|
| **GENERAL COMMENTS** | The comments raised in the last cycle have been addressed. |

| | |
|---|---|
| **REVIEWER NAME** | Yang, Ling |
| **REVIEWER AFFILIATION** | The University of Iowa Roy J and Lucille A Carver College of Medicine, Department of Anatomy and Cell Biology |

| | |
|---|---|
| **REVIEWER CONFLICT OF INTEREST** | No |
| **AI DISCLOSURE** | No, I have not used AI |
| **DATE REVIEW RETURNED** | 21-May-2025 |

| | |
|---|---|
| **GENERAL COMMENTS** | The author has thoroughly addressed the comments raised by this reviewer. |
